# The interplay of DNA methyltransferases and demethylases with tuberization genes in potato (*Solanum tuberosum* L.) genotypes under high temperature

**DOI:** 10.3389/fpls.2022.933740

**Published:** 2022-08-16

**Authors:** Madhushree Dutta, Vidhi Raturi, Vijay Gahlaut, Akhil Kumar, Paras Sharma, Vipasha Verma, Vijai Kishor Gupta, Salej Sood, Gaurav Zinta

**Affiliations:** ^1^Biotechnology Division, CSIR-Institute of Himalayan Bioresource Technology, Palampur, India; ^2^Academy of Scientific and Innovative Research (AcSIR), Ghaziabad, Uttar Pradesh, India; ^3^Agrotechnology Division, CSIR-Institute of Himalayan Bioresource Technology, Palampur, India; ^4^ICAR-Central Potato Research Institute, Modipuram, Uttar Pradesh, India; ^5^Division of Crop Improvement, ICAR-Central Potato Research Institute, Shimla, Himachal Pradesh, India

**Keywords:** DNA methylation, epigenetics, climate change, tuberization, heat stress, thermotolerance

## Abstract

Potato is a temperate crop consumed globally as a staple food. High temperature negatively impacts the tuberization process, eventually affecting crop yield. DNA methylation plays an important role in various developmental and physiological processes in plants. It is a conserved epigenetic mark determined by the dynamic concurrent action of cytosine-5 DNA methyltransferases (*C5-MTases*) and demethylases (*DeMets*). However, *C5-MTases* and *DeMets* remain unidentified in potato, and their expression patterns are unknown under high temperatures. Here, we performed genome-wide analysis and identified 10 C5-MTases and 8 DeMets in potatoes. Analysis of their conserved motifs, gene structures, and phylogenetic analysis grouped *C5-MTases* into four subfamilies (*StMET, StCMT3, StDRM*, and *StDNMT2*) and *DeMets* into three subfamilies (*StROS, StDML*, and *StDME*). Promoter analysis showed the presence of multiple cis-regulatory elements involved in plant development, hormone, and stress response. Furthermore, expression dynamics of *C5-MTases* and *DeMets* were determined in the different tissues (leaf, flower, and stolon) of heat-sensitive (HS) and heat-tolerant (HT) genotypes under high temperature. qPCR results revealed that high temperature resulted in pronounced upregulation of *CMT* and *DRM* genes in the HT genotype. Likewise, demethylases showed strong upregulation in HT genotype as compared to HS genotype. Several positive (*StSP6A* and *StBEL5*) and negative (*StSP5G, StSUT4*, and *StRAP1*) regulators are involved in the potato tuberization. Expression analysis of these genes revealed that high temperature induces the expression of positive regulators in the leaf and stolon samples of HT genotype, possibly through active DNA demethylation and RNA-directed DNA methylation (RdDM) pathway components. Our findings lay a framework for understanding how epigenetic pathways synergistically or antagonistically regulate the tuberization process under high-temperature stress in potatoes. Uncovering such mechanisms will contribute to potato breeding for developing thermotolerant potato varieties.

## Introduction

Global climate change is a serious threat to sustainable crop production. The global mean surface air temperature is expected to rise from 0.5°C to 4.5°C by the end of the twenty-first century (Change, 2013), which is predicted to result in a 17–24% loss in crop productivity (Zhou et al., 2019). The temperate crops are more vulnerable to global temperature rise, negatively affecting their development and final harvest (Lesk et al., 2016).

Potato is a temperate staple food crop with global production of 370.43 million tons (FAOSTAT, 2021). It is one of the richest sources of carbohydrates, fibers, vitamins, and antioxidants (Beals, 2019), feeding the global nutritional requirements (Birch et al., 2012). Potato is vegetatively propagated through tubers, which develop from underground stems known as stolons (Zierer et al., 2021). The stages of tuberization involve different phases, *viz*. stolon induction, elongation/branching, termination of stolon longitudinal growth, tuber induction by sub-apical stolon swelling, tuber growth, and bulking (Vreugdenhil and Struik, 1989).

Tuberization is a complex biological phenomenon affected by several environmental cues (Dutt et al., 2017). Short days, cool temperature, and low nitrogen supply favors tuberization, whereas long days, high temperature, and high nitrogen supply oppose the tuberization process (Gao et al., 2014). The optimal temperature requirement for tuberization is 15–20°C (Rykaczewska, 2015). High temperatures and long days impede the tuberization process (Dutt et al., 2017). The process of tuberization under high temperature involves altered assimilate partitioning and change in the source to sink balance (Hastilestari et al., 2018). Even a slight increase in temperature (e.g., 30°C Day/20°C night) can impact the process of tuberization, affecting crop yield severely (Hancock et al., 2014; Kim et al., 2017). Additionally, phytohormones are involved in the tuberization process. The high GA levels impair tuber development, whereas ABA, cytokinin, and sucrose promote tuberization (Chatterjee et al., 2007). The tuberization signaling network involves several key players, including promoters (*StSP6A, StBEL5, StPOTH1*, and *StCDF*) and inhibitors (*StSUT4, StSP5G*, and *StRAP1*) of tuberization (Dutt et al., 2017).

DNA methylation is a conserved epigenetic mark that regulates gene expression, transposable elements, and stress-mediated responses, eventually contributing to genomic stability (Zhang et al., 2018). DNA methylation in eukaryotes is determined by the concurrent activity of methyltransferases and demethylases (Zhang et al., 2018). In plants, DNA methylation occurs at cytosine residues in three sequence contexts: CpG, CpHpG, and CpHpH (H depicts A, C, T; Law and Jacobsen, 2010). DNA methylation is broadly categorized into *de novo* and maintenance methylation (Zhang et al., 2018). The basic function of any C5-MTase is to recognize specific nucleotide sequences and mark the transfer of methyl group from co-factor S-adenosyl-L methionine (SAM) to 5'-cytosine residue on the pyrimidine ring (Cheng et al., 1993). This function is attributed to the N-terminal regulatory and C-terminal catalytic domain (Pavlopoulou and Kossida, 2007). Plant cytosine DNA methylases (C5-MTase) are much more diverse and less explored than mammalian C5-MTase. Till now, four major types of C5-MTases have been identified including METHYLTRANSFERASE (MET), CHROMO METHYLTRANSFERASE (CMT), DOMAINS REARRANGED METHYLASE (DRM), and DNA METHYLASE HOMOLOG 2 (DNMT2; Cao et al., 2003). DNA demethylation occurs either passively by loss of maintenance methylation enzymes during consecutive DNA replication or actively by the action of a special set of DNA glycosylases (HhH-GPD); DNA glycosylases/lyases that excise 5-meC and operate through the base excision pathway (Roldán-Arjona et al., 2019). Also, two additional conserved domains named RNA recognition motif (RRM) and methylated CpG discriminating CXXC domain are found (Iyer et al., 2011). Thus, active DNA demethylation requires a set of DNA demethylases to initiate the process (Gallego-Bartolomé et al., 2018; Liu et al., 2021).

The concurrent action of methyltransferase and demethylases regulate several developmental features such as plant size, leaf size and shape, flowering time, and fruit ripening (Finnegan et al., 1996). Also, methyltransferases and demethylases are involved in regulating stress-responsive genes in plants (Zhu et al., 2020). For instance, the role of DNA methylation has been suggested in drought and salinity stress responses while studying the contrasting rice genotypes (Garg et al., 2015). Likewise, the role of DNA methylation in conferring plant immunity in *A. thaliana* has also been demonstrated (Dowen et al., 2012).

In the present study, DNA methyltransferase and demethylases were characterized in potato (*Solanum tuberosum*) for the first time. Also, their expression patterns were determined in several tissues such as the leaf, flower, and stolon in the heat-sensitive (HS) and heat-tolerant (HT) potato genotypes under high temperature. Furthermore, the expression of tuberization genes and heat shock factors (HSFs) were studied in these genotypes to delineate how epigenetic components are linked with tuberization under high temperature.

## Materials and methods

### Collection of candidate genes

Protein sequences were downloaded from the potato genome database (Spud DB; Hirsch et al., 2014) by using Pfam ID (PF00145, PF15628, PF00730, and PF15629) to identify the C5-MTases and demethylases. The resultant protein sequences were also verified by downloading the hidden Markov model (HMM) file from the HMMER search and identified through the ENSEMBL database (Potter et al., 2018). The identified potato C5-MTase and demethylase protein sequences were then classified based on the presence of conserved domain using available online software SMART (Schultz et al., 2000) and NCBI_CDD (Marchler-Bauer et al., 2015). Domain Graph 2.0 (DOG 2.0) was used to construct the figures of conserved domains of each class of proteins (Ren et al., 2009). The ExPASy server (http://www.expasy.org/) (Artimo et al., 2012) was used to predict the relative molecular weight (MW) and isoelectric point (PI) of potato C5-MTase and demethylase.

### Analysis of conserved motif and gene structure

The conserved motifs of C5-MTase and demethylases were analyzed using MEME suite vs 5.05 (Bailey et al., 2009), and a total of 15 conserved motifs were identified which was further constructed using TBtool (Chen et al., 2018). Gene Structure Display Server (GSDS 2.0) was used to draw the gene structure of potato C5-MTase and demethylase (Hu et al., 2015).

### Identification of promoter cis-regulatory elements

The sequence retrieval tab of the potato genome database (Spud DB) was used to extract 2 kb sequences that lie upstream of the Transcription Start Site (TSS) of genomic sequences of C5-MTases and demethylases. The online software PlantCare (Lescot et al., 2002) was used to list out the conserved elements of promoter sequences.

### Analysis of multiple sequence alignment and phylogenetic tree

Full-length amino acid sequences of C5-MTase (*Arabidopsis thaliana, Solanum lycopersicum, Solanum tuberosum, Glycine max, Zea mays*, and *Oryza sativa*) and DNA demethylase (*Arabidopsis thaliana, Solanum lycopersicum, Solanum tuberosum, Cucumis sativa*, and *Glycine max*) were subjected to multiple sequence alignments by using muscle program of MEGA X (10.05) software (Kumar et al., 2018). The aligned protein sequences were used to construct a phylogenetic tree, using the Neighbor-Joining (NJ) program for 1,000 bootstrap replicates with Poisson correction (Yang, 2007).

### Prediction of subcellular localization

The subcellular localization of candidate genes was determined by using the protein sequences in the web tool CELLO v.2.5 (Yu et al., 2014). The localization of genes was further used to map them.

### Experimental set-up

Heat-sensitive (Kufri Chandramukhi, CP2141/A-2708) and -tolerant (Kufri Kiran, CP4803/A-2708) potato tubers were obtained from the Central Potato Research Institute (CPRI), Shimla, India. Well-sprouted tubers were grown in 0.2 L and 12 cm pots containing soil mixture (soil: vermicompost:sand, 2:1:1). Six replicates per genotype were raised in a Percival growth chamber under 16 h light and 8 h darkness conditions with day and night temperatures of 22 and 18°C, respectively. The light intensity of the growth chamber was 300 μmol/s/m^2^. The plants were watered and rotated daily to avoid water deficit and minimize positional effects. After 50 days, half of the plants were subjected to 14 days high-temperature stress (32°C day and 27°C night). The leaves, flowers, and stolon tissues were harvested from both the control and heat treatments around 10 AM (4 h of light onset). The harvested tissues, *viz*. leaf, flower, and stolon of similar size/age, were collected in aluminum zippers, frozen immediately in liquid nitrogen, and stored at −80°C.

### RNA isolation, cDNA synthesis, and quantitative real-time PCR (qPCR)

About 100 mg of the frozen tissue was finely ground with a mortar and pestle (Yu et al., 2007). RNA was isolated with Spectrum Plant Total RNA Kit (Sigma Aldrich, USA) by following the manufacturer's protocol. The isolated RNA was treated with 1 U/μl of DNase I (ThermoFisher Scientific Baltics, UAB, Lithuania). Further, 1 μg of RNA was used to synthesize cDNA using the Verso cDNA synthesis kit (ThermoFisher Scientific Baltics, UAB, Lithuania). qPCR primers were designed from the CDS sequence using the ApE software (Davis and Jorgensen, 2022) (Supplementary Table S1). The quantitative real-time PCR was carried out using the Dynamo Colorflash SYBR Green (ThermoFisher Scientific Baltics, UAB, Lithuania) in an Applied Biosystem step-plus real-time PCR system. Three biological and three technical replicates were used to perform qPCR analysis. The expression of the housekeeping gene tubulin was found to be stable across tissues, treatments, and genotypes. Hence, tubulin was used as an internal control to measure target gene expression using the 2–ΔΔCt method (Schmittgen and Livak, 2008).

### Statistical analysis

The gene expression data obtained by qPCR were expressed as mean ± standard error (SE) for three biological and technical replicates. Statistical analysis was carried out by using the SPSS 16.0 software (SPSS Science, UK). The significant difference between control and heat-treated plants was analyzed based on two-tailed Student's *t*-test. The significant difference between control and heat-treated samples at *p* < 0.05, *p* < 0.01, and *p* < 0.001 are denoted by one, two, and three asterisks.

## Results

### Gene identification, structure analysis, and chromosome localization of *C5-MTase* and *demethylase* genes

**Figure 1:**
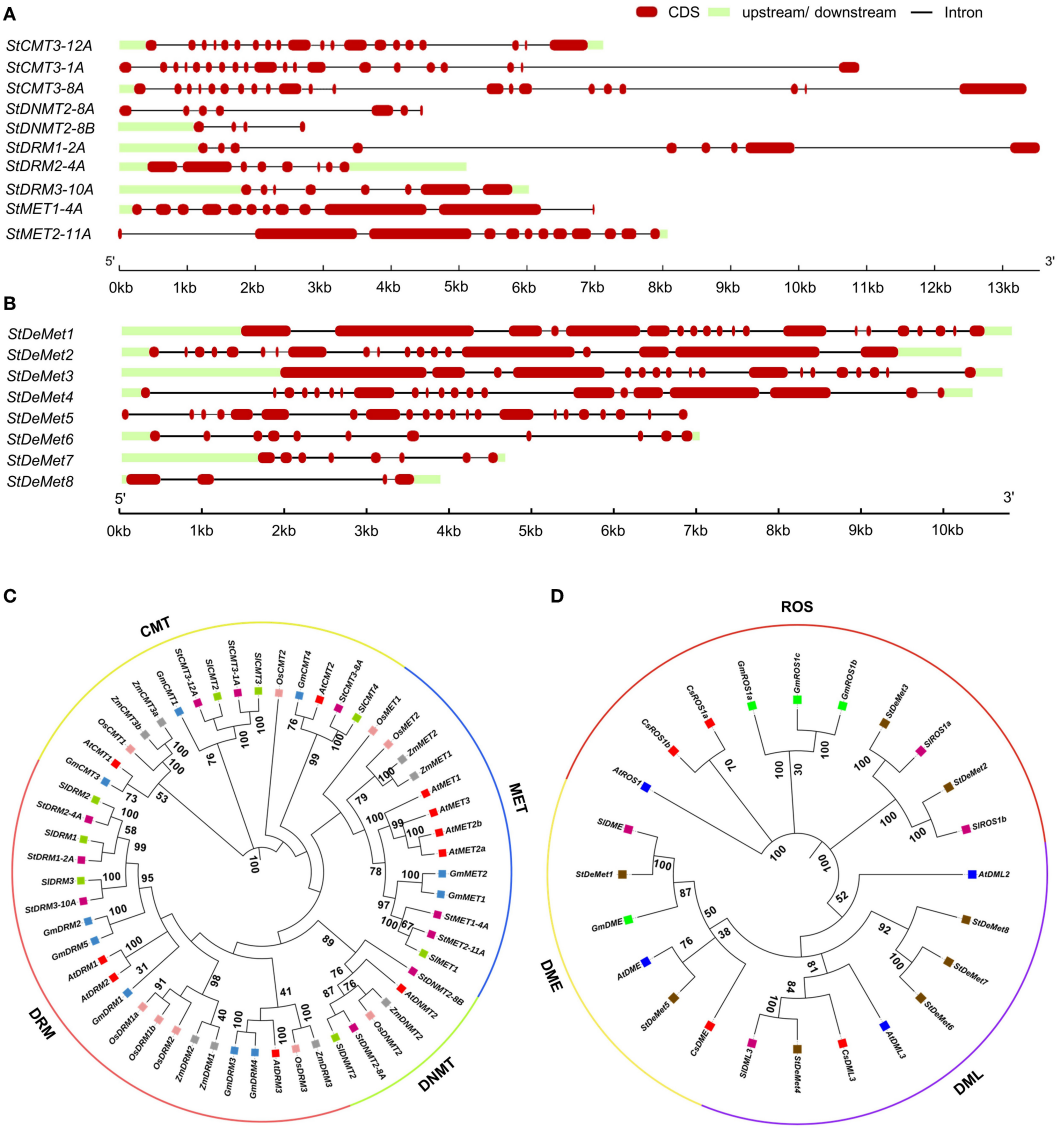
Gene structures of *StC5-MTase* **(A)** and *StDeMets* **(B)** in *S. tuberosum*. Phylogenetic tree analysis of C5-MTase **(C)** and DeMet proteins **(D)** with 1,000 bootstrap replicates. The lines in gene structure represent introns and filled red and light green boxes represent CDS and UTR, respectively. The lengths of CDS and introns can be determined using the scale bar on the bottom.

**Figure 2:**
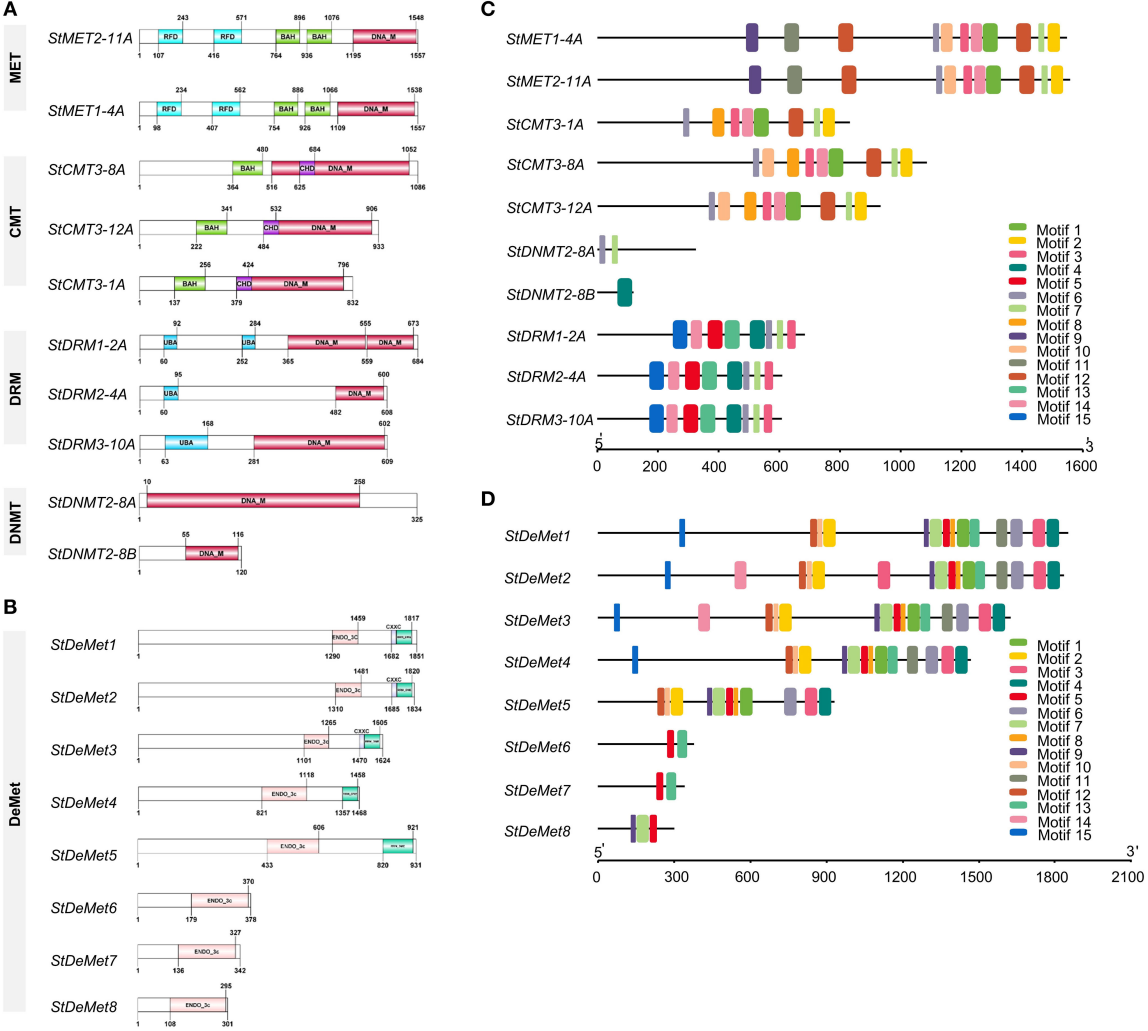
Schematic structures of conserved domains present in *StC5-MTase* **(A)** and *StDeMet* **(B)** proteins. The conserved motifs present in C5-MTase **(C)** and DNA demethylase **(D)** genes in *Solanum tuberosum*. (i) The conserved domains in the CMT family possess CHROMO (Chromatin organization modifier) and one BAH (Bromo Adjacent Homology); (ii) MET has two BAH and an RFD domains; (iii) DRM family has a unique UBA domain (Ubiquitin-associated); whereas (iv) DNMT has shorter protein length due to lack of N-terminal regulatory domain. *StDeMet* family possesses DNA glycosylase (ENDO_3C), RNA Recognition Motif (RRM), and methylated CpG discriminating (CXXC). The color boxes in the motif represent the position of different motifs and box sizes show the length (aa) of motifs.

In our present study, we identified 10 C5-MTase (*StMET1-4A, StMET2-11A, StCMT3-1A, StCMT3-8A, StCMT3-12A, StDNMT2-8A, StDNMT2-8B, StDRM1-2A, StDRM2-4A*, and *StDRM3-10A)* and 8 DNA demethylase genes (*StDeMet1, StDeMet2, StDeMet3, StDeMet4, StDeMet5, StDeMet6, StDeMet7*, and *StDeMet8)*. The CDS and protein length of methyltransferase genes varied from 363 bp (*StDNMT2-8B)* to 4,674 bp *(StMET2-11A)* and 120 amino acids (aa) (*StDNMT2-8B)* to 1,557 aa *(StMET2-11A)*, respectively. Similarly, gene transcript length and polypeptide length of demethylase genes ranges from 906 bp (*StDeMet8)* to 5,556 bp *(StDeMet1)* and 301aa (*StDeMet8)* to 1,851 aa *(StDeMet1)*, respectively (Figure 1). The predicted molecular weight and pI value range from 174.27 to 13.7 kDa and 4.63 to 9.51 in C5-MTase; and 207.46 to 33.51 kDa and 6.24 to 9.46 in DeMets (Table 1). The chromosome localization of the candidate proteins has also been incorporated (Table 1). Interestingly, the classification of C5-MTase is based on the linear arrangement of distinct and conserved domains across members of a different enzyme family (Pavlopoulou and Kossida, 2007). Pfam analysis revealed that all C5-MTases (PF00145) have conserved DNA_Methylase (DNA_M) domain. However, different C5-MTase are characterized due to the presence of distinctive domains at their N-terminal: (i) CMT family possesses CHROMO (Chromatin organization modifier) and one BAH (Bromo Adjacent Homology) in contrast to (ii) MET which has two BAH and an RFD domains (iii) DRM family has unique UBA domain (Ubiquitin-associated), whereas (iv) DNMT has shorter protein length as compared to other C5-MTase owing to lack of N-terminal regulatory domain (Figure 2). Additionally, DeMets have conserved HhH-GPD (PF00730), Perm-CXXC (PF15629), and RRM_DME (PF15628). The distribution of conserved motifs in the StC5-MTase and StDeMets proteins were predicted using the MEME online suite. A total of 15 conserved motifs were explored in StC5-MTase and StDeMets proteins. Motifs 9, 11, and 12 were highly conserved in the MET subfamily. Motifs 5, 13, and 15 were major motifs in DRM, while Motif 8 was unique to CMT3 subfamilies. The motif distribution in StDeMets proteins showed that Motifs 1, 3, 4, 5, 6, 7, 8, 9, 11, and 13 were highly conserved in StDeMet 1- 5 proteins, while DeMet 2 and 3 have additional unique Motif 14 (Figure 2). The structure of *StC5-MTase* and *StDeMets* of potato is identical to *Arabidopsis thaliana* and is presumed to catalyze similar functions as that of *Arabidopsis thaliana* in potato.

**Table 1:** Basic information of C5-MTase and DNA demethylase genes in *Solanum tuberosum*.

		**Chromosome**						**CDS**
**Protein name**	**Gene ID**	**number**	**Chromosome location**		**Protein**			**length (bp)**
**Cytosine-5 DNA methyltransferases**					**PI**	**Mw (kDa)**	**Length (aa)**	
*StMET1-4A*	Soltu.DM.04G014710	Chr04	23868453	23875446	6.15	174.27	1547	4644
*StMET2-11A*	Soltu.DM.11G013230	Chr11	16840303	16848391	6.2	175.13	1557	4674
*StCMT3-1A*	Soltu.DM.01G001630	Chr01	1678832	1689723	4.72	94.08	832	2499
*StCMT3-8A*	Soltu.DM.08G003560	Chr08	4677712	4691064	8.96	121.97	1086	3261
*StCMT3-12A*	Soltu.DM.12G000130	Chr12	355235	362351	5.25	104.5	933	2802
*StDNMT2-8A*	Soltu.DM.08G015570	Chr08	43136073	43140542	5.03	37.11	325	978
*StDNMT2-8B*	Soltu.DM.08G015580	Chr08	43143967	43146720	9.51	13.7	120	363
*StDRM1-2A*	Soltu.DM.02G006560	Chr02	20245172	20258718	4.8	77.1	684	2055
*StDRM2-4A*	Soltu.DM.04G000230	Chr04	452787	457895	4.63	68.47	608	1827
*StDRM3-10A*	Soltu.DM.10G030090	Chr10	60637223	60643251	5.12	69.03	609	1830
**DNA demethylases**								
*StDeMet1*	Soltu.DM.11G005260	Chr11	5686999	5397802	7.47	207.46	1851	5556
*StDeMet2*	Soltu.DM.09G004240	Chr09	3690956	3701147	6.24	204.72	1834	5505
*StDeMet3*	Soltu.DM.10G024770	Chr10	56407648	56417613	6.63	181.56	1624	4875
*StDeMet4*	Soltu.DM.03G037240	Chr03	60022718	60033043	7.76	165.45	1468	4407
*StDeMet5*	Soltu.DM.04G021680	Chr04	49931927	49938794	9.23	105.71	931	2796
*StDeMet6*	Soltu.DM.06G028750	Chr06	53964389	53971402	9.46	41.5	378	1137
*StDeMet7*	Soltu.DM.09G025490	Chr09	61584476	61589128	9.17	33.8	299	900
*StDeMet8*	Soltu.DM.03G027780	Chr03	52412404	52416269	8.31	33.51	301	906

### Phylogenetic analysis

To exemplify the evolutionary relationship among C5-MTase subfamilies in plants, we used 55 protein sequences from four dicots (*Arabidopsis thaliana, Solanum lycopersicum, Solanum tuberosum*, and *Glycine max*) and two monocots (*Zea mays* and *Oryza sativa*) to construct a phylogenetic tree (Supplementary Table S2). C5-MTase is grouped into four subfamilies: DRM, CMT, MET, and DNMT having 21, 15, 13, and six members, respectively (Figure 1). Subsequently, the largest and smallest group in the tree were DRM and DNMT subfamily. Further, the subfamilies could also be divided into monocot and dicot distinct subgroups, based upon the significant differences in protein sequences. Interestingly, in almost all clades, tomato C5-MTase was closer to potato C5-MTase protein, suggesting that they are the members of the same taxonomic family, *Solanaceae*, and predicted to share functional similarity.

As for DNA Demethylases, 24 protein sequences from four dicots (*Arabidopsis thaliana, Solanum lycopersicum, Solanum tuberosum and Cucumis sativa*, and *Glycine max*) were extracted to construct a phylogenetic tree (Supplementary Table S3) arranged in ascending order in accordance to the number of members: DME, DML, and ROS. However, we analyzed *DeMets* from dicots as DME evolved monophyletically in dicots, such results are consistent with the previous report (Yu et al., 2021). We also observed DML group constituting four out of eight *StDeMets* genes as compared to ROS and DME.

### Analysis of regulatory elements in the promoter region

**Figure 3:**
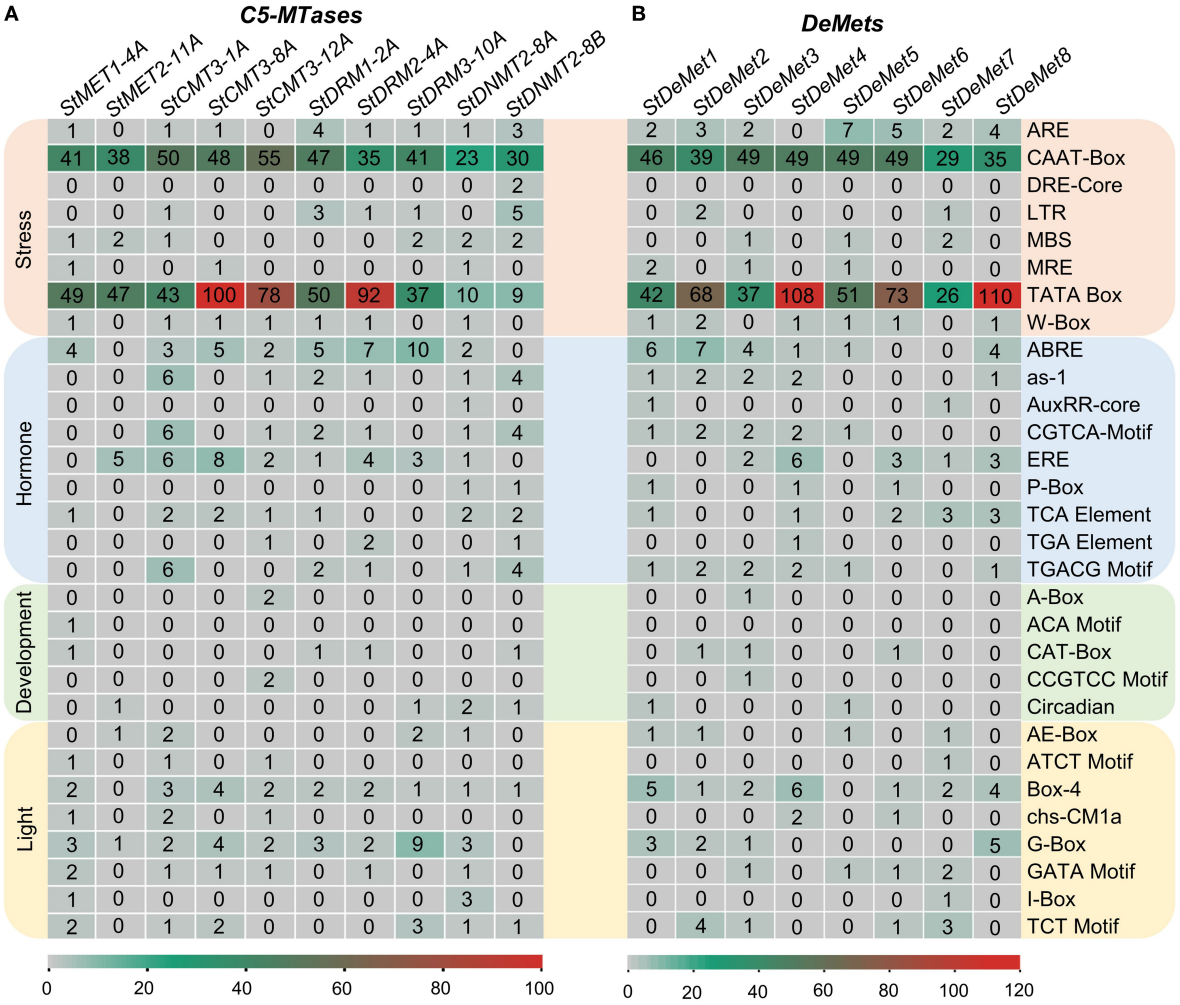
Cis-acting regulatory elements in the promoters of **(A)** *StC5-MTase* and **(B)** *StDeMets*. Cis-regulatory elements are classified into four categories (light, development, hormones and stress responses). The numbers in the heat map depict the abundance of cis-regulatory elements in the promoter region.

Cis-regulatory elements are the core components governing biological processes. A sum of 30 major elements was represented that were predicted in the 2 kb upstream of the TSS in the promoter region. They were further categorized into light, development, hormone, and stress-responsive cis elements (Figure 3). C5-MTase genes showed several stress-responsive cis elements, such as CAAT and TATA box, which were predominant across the subfamilies/members (MET, CMT3, DRM, and DNMT2). Among all the subfamilies, CMT3 had the largest number of stress-involved elements. We found nine hormone-responsive elements such as ABRE and ERE that were abundant in all members. However, AuxRR-core is present exclusively in *StDNMT2-8A*. Elements involved in plant development were also screened, and five major elements including A box, CCGTCC, CAT box, ACA motif, and Circadian were identified. A box and CCGTCC were present only in *StCMT3-12A*, while circadian control element and ACA motif were found exclusively in *DNMT2* and *MET* subfamilies. Further, eight CRE elements of the light-responsive module were also identified, out of which G box and Box 4 were found in all the naturally grouped subfamilies.

Similarly, cis-regulatory elements were also analyzed in the potato DNA methyltransferase proteins. ARE, CAAT, and TATA control elements were present in all the eight *DeMet* genes. Surprisingly, the abscisic hormone element ABRE was not found in *StDeMet6* and *StDeMet7*, while TCA elements involved in salicylic acid response were present in *StDeMet6* and *StDeMet7*. Furthermore, light module Box4 was present in all members. Altogether, our results suggest that C5-MTase and demethylase genes are involved in plant growth, development, and various abiotic stress responses.

### Sub-cellular localization of potato C5-MTase and DeMets

**Figure 4:**
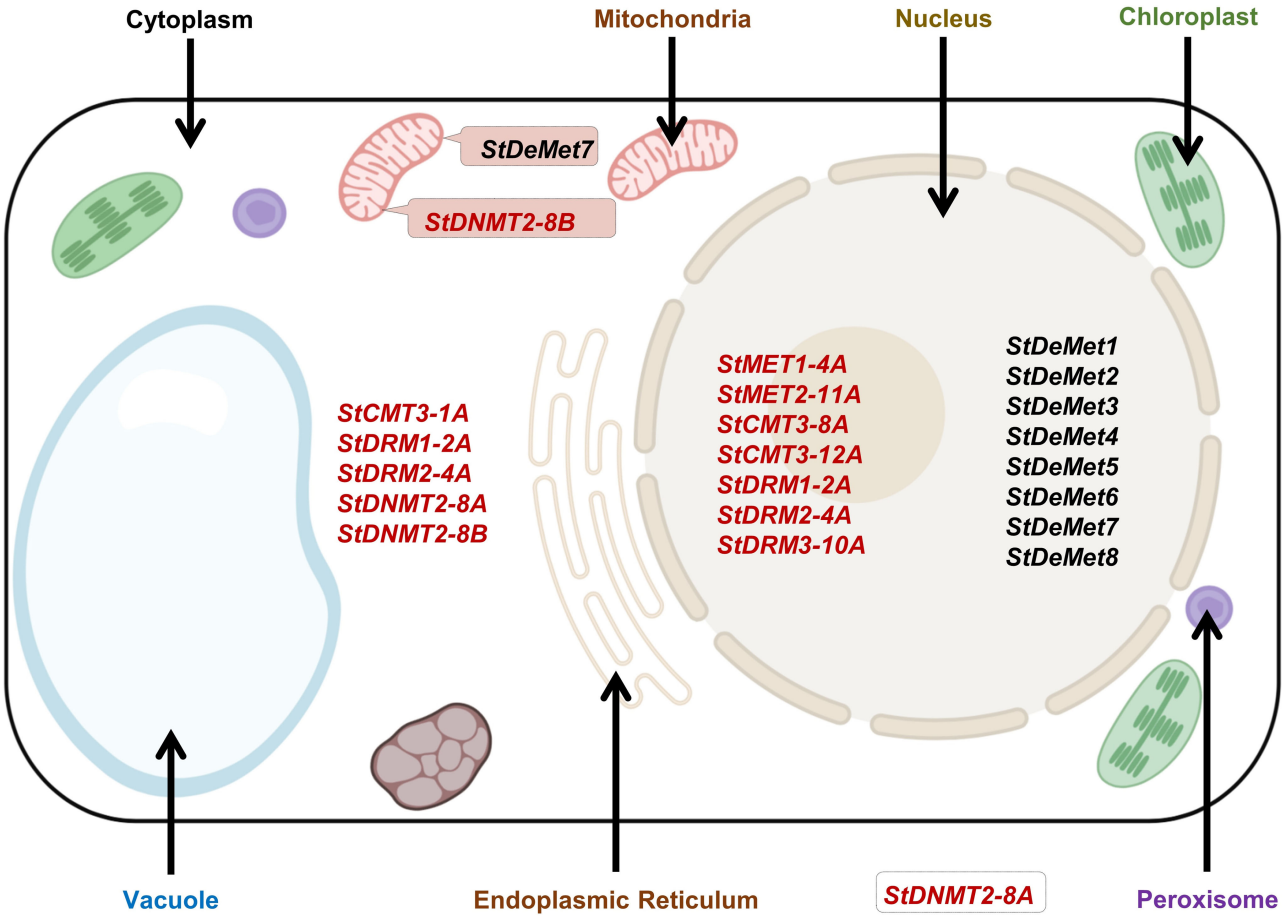
Subcellular localization of StC5-MTase and StDeMet proteins in potato predicted using the CELLO program. Partially adapted from Gahlaut et al. (2020).

The *in silico* subcellular localization revealed that all DeMets were localized in the nucleus; however, *StDeMet7* was additionally found in mitochondria. The C5-MTases were localized in the nucleus (*StMET1-4A, StMET2-11A, StCMT3-8A, StCMT3-12A, StDRM1-2A, StDRM2-4A*, and *StDRM3-10A)* and cytoplasm (*StCMT3-1A, StDNMT2-8A*, and *StDNMT2-8B)*. Apart from being in the nucleus, a couple of DRM members (*StDRM1-2A* and *StDRM2-4A)* were also localized in the cytoplasm (Figure 4).

### Expression patterns of *C5-MTase* and *DeMet* genes

**Figure 5:**
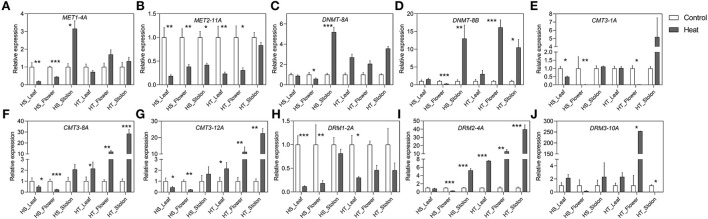
**(A–J)** Relative expression levels of potato C*5-MTase* genes in different tissues in heat sensitive (HS) and tolerant (HT) genotypes under high-temperature stress. Three biological samples were measured with three technical replicates. The fold change in RNA levels was calculated as the 2–ΔΔCt value relative to the mean values obtained in the leaf, flower and stolon samples (set at a value of 1.0). Standard errors of the means are shown with one, two and three asterisks indicating significant differences (*p* < 0.05, *p* < 0.01, *p* < 0.001, respectively) using a student's *t*-test.

**Figure 6:**
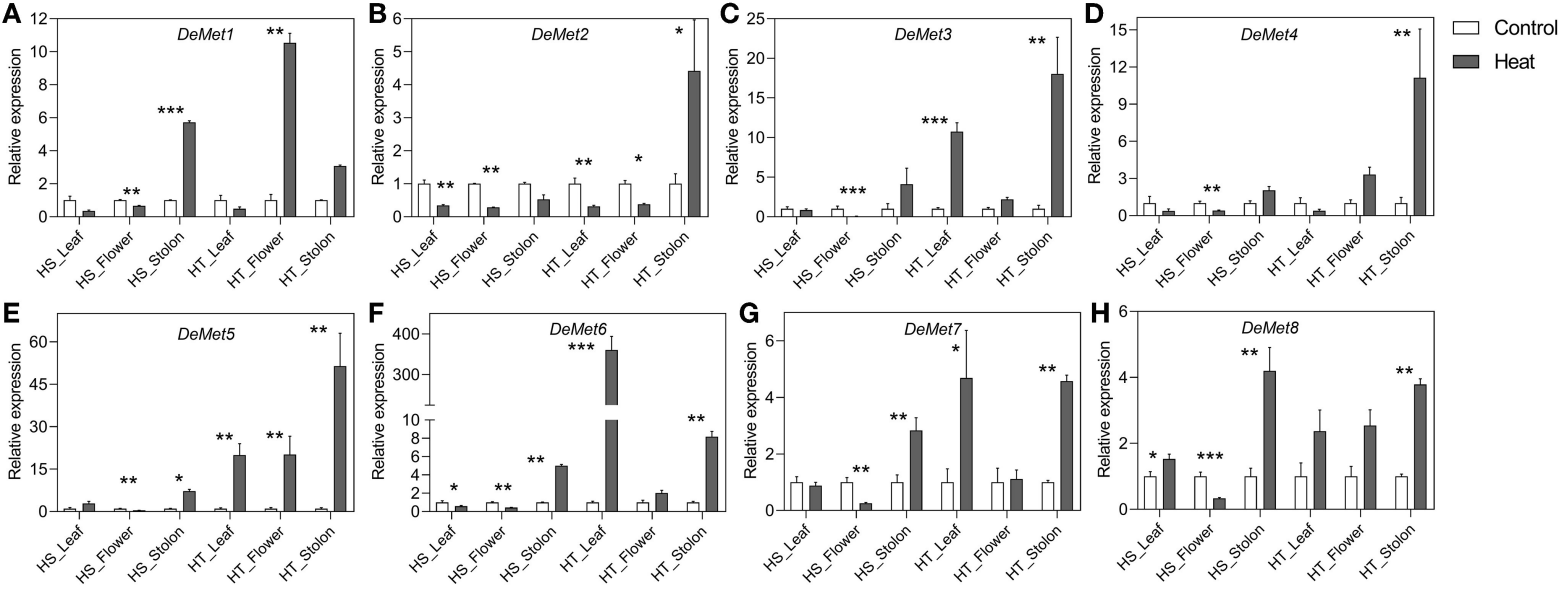
**(A–H)** Relative expression levels of potato DeMet genes in different tissues in heat sensitive (HS) and tolerant (HT) genotypes under high temperature stress. Three biological samples were measured with three technical replicates. The fold change in RNA levels was calculated as the 2–ΔΔCt value relative to the mean values obtained in the leaf, flower and stolon samples (set at a value of 1.0). Standard errors of the means are shown with one, two and three asterisks indicating significant differences (*p* < 0.05, *p* < 0.01, *p* < 0.001, respectively) using a student's *t*-test.

The expression dynamics of *C5-MTase* and *DeMet* genes were studied in different tissues, *viz*. leaf, flower, and stolon in the heat-sensitive (HS) and heat-tolerant (HT) potato genotypes under high temperature (Figures 5, 6). The expression of *StMET1-4A* and *StMET2-11A* showed a significant decline both in leaf and floral tissues in heat-sensitive genotype as compared to heat tolerant. However, stolon tissue shows upregulation and downregulation of *StMET1-4A* and *StMET2-11A*, respectively, in heat-sensitive cultivar. *StDNMT-8A* and *StDNMT-8B* expression level is predominantly detected in stolon tissues of HS. Moreover, heat induced the expression of *StDNMT-8B* in the flower of HT. The expression profile of the CMT subfamily is significantly higher in HT as compared to HS across three tissues (leaf, flower, and stolon) except *StCMT3-1A*. *StDRM1-2A* is downregulated in HT and HS in all samples. Interestingly, the expression level of *StDRM2-4A* increased in leaves, flowers, and stolon in heat-tolerant genotype. Overall, *C5-MTase* genes respond differently in different tissues of two genotypes, wherein CMT and DRM subfamilies showed pronounced heat-induced upregulation in the tolerant genotype.

*DeMets* are involved in DNA demethylation, and we observed a varied expression pattern across different tissues and genotypes. Heat resulted in the downregulation of *StDeMet1* in flower; however, the stolon tissue showed upregulation in the HS genotype. In contrast, *StDeMet1* showed upregulation in flower. In general, *StDeMet2* expression was downregulated in all the tissues of both the genotypes under heat stress, except the stolon of HT where it showed upregulation. Importantly, the expression of *StDeMet3, StDeMet5, StDeMet6*, and *StDeMet7* was induced under heat in the leaf and stolon tissues of the HT genotype. The expression of *StDeMet4, StDeMet5*, and *StDeMet6* were downregulated in the flower of HS. In the case of *StDeMet8*, HS leaf showed upregulation, while flower showed downregulation; however, the stolon tissue showed upregulation in both the genotypes.

### Expression patterns of tuberization and HSFs genes

**Figure 7:**
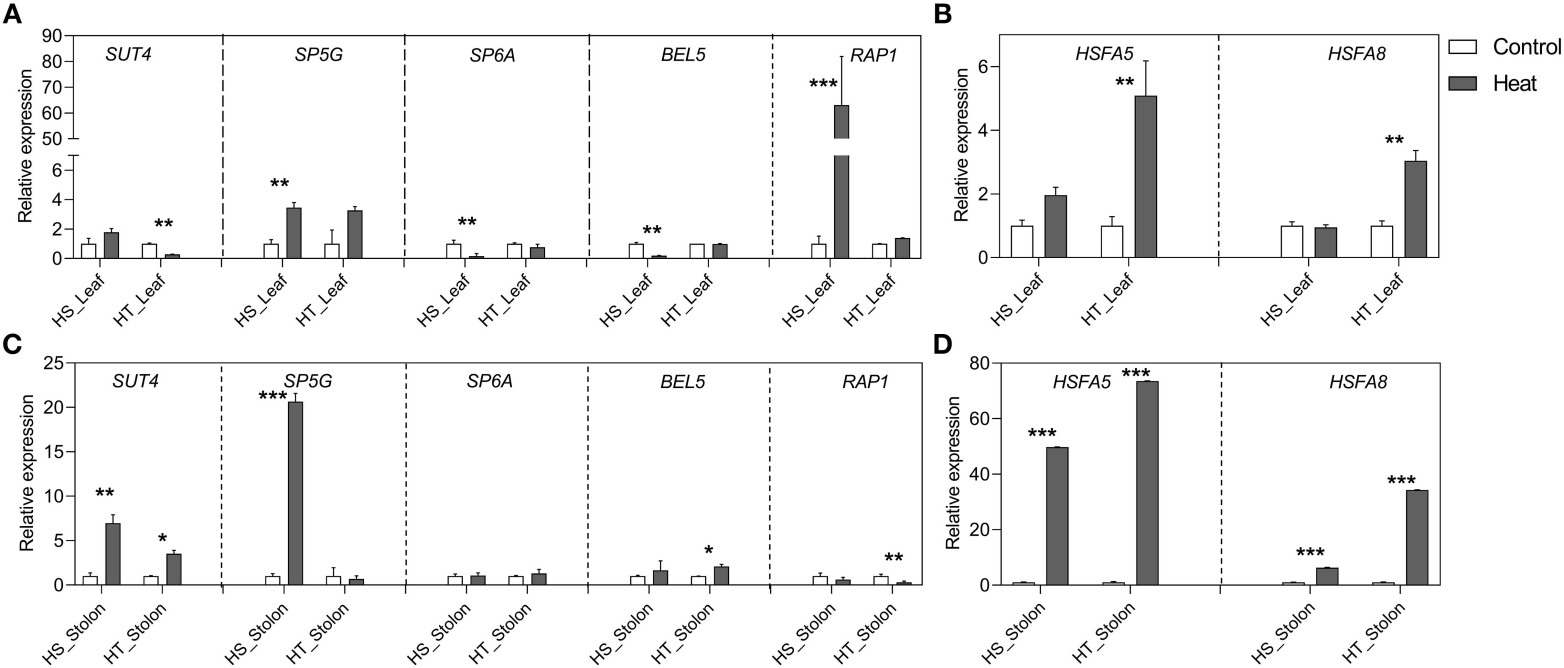
Relative expression levels of potato tuberization genes and HSFs in leaf **(A,B)** and stolon **(C,D)** tissues in heat-sensitive (HS) and -tolerant (HT) genotypes under high-temperature stress. Three biological samples were measured with three technical replicates. The fold change in RNA levels was calculated as the 2–ΔΔCt value relative to the mean values obtained in the leaf and stolon samples (set at a value of 1.0). Standard errors of the means are shown with one, two, and three asterisks indicating significant differences (*p* < 0.05, *p* < 0.01, *p* < 0.001, respectively) using a Student's *t*-test.

Several genes play an important role in tuber formation in potato, wherein the concurrent action of positive (e.g., *StSP6A* and *StBEL5*) and negative regulators (e.g., *StSUT4, StSP5G*, and *StRAP1*) determine the tuber yield. Also, Heat Shock Factors (HSFs) are well-known to impart thermotolerance in plants. Thus, we analyzed the expression of tuberization and HSF genes in the leaf and stolon tissues (Figure 7).

Heat resulted in the downregulation of *StSUT4* expression in the leaf tissues of the HT genotype, whereas *StSP5G* showed significant upregulation in stolon tissues of the HS genotype. *StSP6A* is a master regulator of tuberization that is affected by high-temperature stress. Heat resulted in the significant suppression of *StSP6A* in leaves of HS genotype compared to HT genotype (Figure 7). However, unexpectedly intrinsic basal transcript level was maintained in stolon tissues of HS and HT genotypes. *StBEL5* expression was downregulated by high temperature in the leaf tissue of the HS genotype, while it was stable across leaf and stolon tissues of the HT genotype. *StRAP1* showed enhanced expression in leaves while it remained unaltered in stolons of HS genotype. Furthermore, the expression of HSFs was quantified. We found that *StHSFA5* and *StHSFA8* were strongly upregulated in the HT genotype both in leaves and stolons tissues under heat stress, suggesting its role in thermotolerance.

## Discussion

Potato is a temperate crop that is challenged by several environmental cues such as high temperature. High-temperature exposure alters the partitioning of assimilates and accumulation of biomass in plant organs, which results in stem elongation, an increase in leaf number, and a decline in tuber yield (Wolf et al., 1991; Lafta and Lorenzen, 1995). A complex gene regulatory network comprising FT orthologs SELF PRUNING 6A (*StSP6A*) and SELF PRUNING 5G (*StSP5G*), CONSTANS-LIKE 1 (*StCOL1*), BEL1-like homeodomain (*StBEL5*), and an AP2-like gene (*StRAP1*) plays an important role in potato tuberization (Navarro et al., 2011; Hancock et al., 2014). For example, SP6A is a master regulator that interacts with its FD partner (*StFDL*) and scaffold protein St14-3-3 to form Tuber Activating Complex (TAC) to induce stolon–tuber induction (Hannapel and Banerjee, 2017). Recent developments have established that the *StSP6A* transcript is suppressed under elevated temperature, possibly through another FT homolog, *StSP5G*, which functions to repress tuberization through the accumulation of *StCO*. Similarly the antagonistic role of FT homologs in controlling flowering time in sugar beet has been reported (Pin et al., 2010). Furthermore, high temperature causes *SP6A* repression by a micro-RNA named SUPPRESSING OF SP6A (SES; Lehretz et al., 2019). Also, recently it has been shown that *SP6A* is suppressed transcriptionally and post-transcriptionally under high-temperature stress (Park et al., 2022). Epigenetic components are also involved in potato tuberization, wherein Polycomb Repressive Complex (PRC)-mediated histone modifications repress several target genes by H3K27 trimethylation (Kumar et al., 2021). The role of DNA methylation in photoperiod-mediated tuberization has also been recently reported (Ai et al., 2021). However, DNA methylation and demethylation enzymes remain uncharacterized in potatoes and their role in potato tuberization under high temperature is unexplored. The concurrent action of DNA methyltransferase and demethylase carries out DNA methylation and demethylation respectively (Lang et al., 2017).

In the present study, we identified several *C5-MTases* and *DeMets* in potatoes and studied their expression dynamics in the different tissues of the two contrasting genotypes, *viz*. Kufri Chandramukhi (heat sensitive) and Kufri Kiran (heat tolerant) under high temperature stress. We found 10 *C5-MTase* and 8 *DeMets* in potato, and several C5-MTase and DeMets were also reported in other allotetraploid soybean and peanut (Song et al., 2013; Wang et al., 2016), which is comparatively higher than the diploid species (Kong et al., 2020). This suggests that polyploidization events potentially contribute to the enhancement of plant epigenetic factors. Furthermore, based on their domain structure and phylogenetic analysis, C5-MTases were divided into four different subfamilies, *viz*. METs, CMTs, DRMs, and DNMTs. The members within the same subfamily shared similar domain architecture and exon-intron boundary, but it differed between sub-families. Our results are consistent with the previous reports on *Solanum lycopersicum* (Cao et al., 2014). The principal maintenance of symmetric CG methylation is operated by METs, an ortholog of mammalian DNMT1 (Zhang et al., 2018). Contrastingly, METs in plants cannot distinguish hemimethylated CG dinucleotides from non-methylated CG, unlike DNMT1, as it lacks a cysteine-rich CXXC domain (Song et al., 2011). BAH domain of METs facilitates protein-protein interactions, which result in inhibition of gene expression and establish an interconnection among cytosine methylation, replication, and transcription (Callebaut et al., 1999). CMTs are involved in maintaining CHG methylation in centromeric and transposable regions (Gehring and Henikoff, 2008). The chromodomain of CMTs is responsible for protein association with heterochromatin (Gehring and Henikoff, 2008). The protein structure of maize revealed the association of chromo and BAH domain with H3K9me2 and shed light on the functional activity of CMTs (Stroud et al., 2014). The maintenance of “non-symmetrical” CHH methylation is catalyzed by DRMs that operates through the RNA-directed DNA methylation pathway (RdDM). The N-terminal UBA domain provides a connecting link between DNA methylation and ubiquitin-mediated protein degradation. This domain also promotes the degradation of DRMs at specific points in the cell cycle (Cao et al., 2000).

*DeMets* in potato also has conserved domains that are responsible for functional identity and activity (Zhang et al., 2018). The functional demethylases contain HhH-GPD (Endo_3c) domain that is responsible for cleaving glycosidic bonds and generating AP sites (Gehring and Henikoff, 2008). Sometimes, two additional conserved domains named RNA recognition motif (RRM) and methylated CpG discriminating CXXC domain are also found (Iyer et al., 2011). The evolutionary studies revealed that *DeMets* expression varied across species of different genotypes (Yang et al., 2019).

The sub-cellular localization of any protein is important for determining its function in plants. Therefore, we performed *in-silico* subcellular prediction to identify the location of *C5-MTases* and *DeMets*. *C5-MTases* were localized either in the nucleus or in the cytoplasm. These results are consistent with the previous studies on *Solanum lycopersicum*, which reported the localization of *C5-MTase* in the nucleus and cytoplasm (Cao et al., 2014). The presence of *C5-MTase* in both these organelles indicates its possible involvement in stress management machinery (Gahlaut et al., 2020). The exclusive nuclear localization of *DeMets* suggests its potential role in replication and transcriptional regulation (Wang et al., 2016).

Cis-regulatory elements play an important role in the regulation of biological processes and molecular function under developmental transitions and stress episodes (Yamaguchi-Shinozaki and Shinozaki, 2005). The promoter analysis of potato *C5-MTase* and *DeMets* revealed that cis-regulatory elements are classified into four categories such as light response, development, hormones, and stress responses. The presence of CAAT box in the promoter region plays an essential role in leaf development by modulating jasmonate pathways in proliferating tissues as reported in *Nicotiana benthamiana* (*NbCMT3–2*; Lin et al., 2015). The predominance of TATA box elements in the promoter region confers stress responsiveness, whereas TATA-less genes are related to cell growth or housekeeping activity (Bae et al., 2015). The abundance of ABRE and ERE in all the members demonstrates the active role of *C5-MTase* and *DeMets* in abscisic acid and ethylene responsiveness (Kaur and Asthir, 2017). Also, stress-specific regulatory elements such as ARE and MBS were found in the *C5-MTase* of the tea plant (Zhu et al., 2020). Overall, these findings highlight the role of potato's *C5-MTase* and *DeMets* in plant development and stress responses (Gahlaut et al., 2020).

The relationship between DNA methylation and heat stress is less explored. DNA methylation level in different tissues regulates plant growth and development *via* controlling the transcript levels of developmentally important genes (Zhang et al., 2018). We investigated the expression pattern of *C5-MTase* and *DeMets* in two contrasting genotypes across different tissues and observed differential DNA methylation. Our results are similar to studies on rice and rapeseed (Karan et al., 2012; Gao et al., 2014). The decline in expression of *StMET1-4A* and *StMET2-11A* in leaf and floral tissues in heat-sensitive genotype possibly led to reduced CG methylation.

Similarly, Arabidopsis *met1* mutant with reduced MET level showed delayed flowering and juvenile-to-adult transition (Kankel et al., 2003). Such delayed flowering was due to hypomethylation of transposable element (TE) upstream of the *FLOWERING WAGENINGEN* (*FWA*) gene (Kankel et al., 2003). This shows how MET regulates the morphological characteristics and flowering time of plants (Finnegan and Kovac, 2000). We also found that CMT and DRM subfamilies showed pronounced heat-induced upregulation in the tolerant genotype. These findings indicate the role of CMT and DRM in the suppression of TEs (Ramakrishnan et al., 2021). High-temperature stress causes genomic instability and impairs plant health by activating TEs such as *ONSEN* (Chang et al., 2020). Several plant developmental abnormalities have been observed in the DNA methylation-free *C5-MTase* mutants of Arabidopsis, suggesting their role in controlling gene expression and developmental processes in plants (He et al., 2022). However, the reduced expression of CMT and DRM subfamilies in heat-sensitive genotypes across different tissues might be an alternative strategy to recover from heat stress by causing activation of SUPPRESSOR OF DRM1 DRM2 CMT3 (SDC), thus leading to loss of non-CG methylation (Popova et al., 2013). Differential methylation landscapes between genotypes can potentially contribute to stress adaptation (Eriksson et al., 2020).

Heat-induced DNA demethylase gene expression was observed in both genotypes. The expression of the demethylase gene family was more pronounced in HT genotype as compared to the HS genotype. Our results showed that *DeMets-like StDeMet2,StDeMet3, StDeMet4, StDeMet5, StDeMet6*, and *StDeMet7*, were significantly expressed in stolons of HT genotype. Such results are consistent with the increased expression of *ROS* and *DML* like demethylase gene family in Arabidopsis (Hsieh et al., 2009), suggesting its involvement in combating abiotic stress events. Consistently, active DNA demethylation is also associated with the upregulation of stress-responsive genes (Verhoeven et al., 2010).

**Figure 8:**
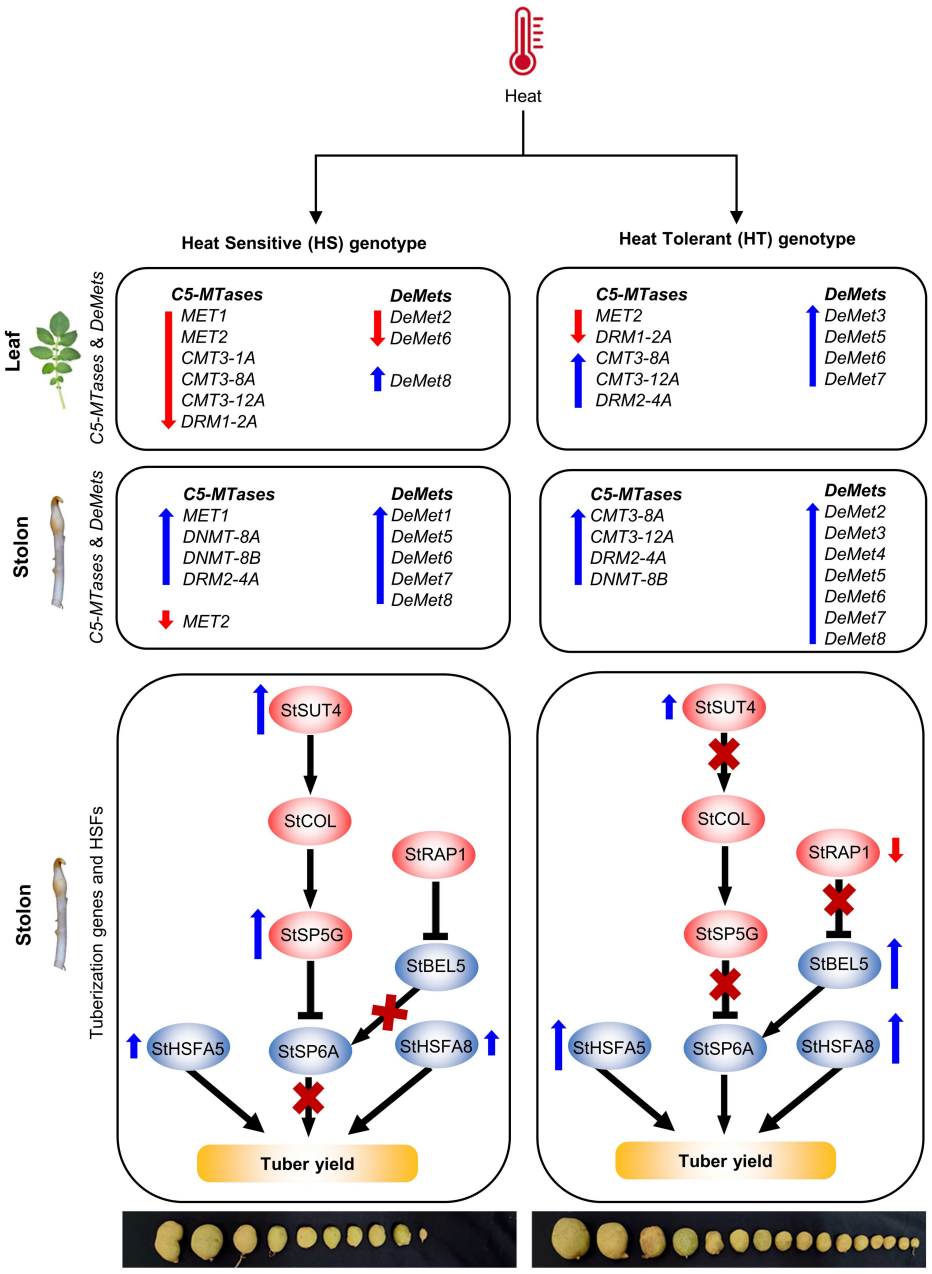
The schematic model depicting the role of *C5-MTase* and *DeMets* gene with potato tuberization genes under high temperature in heat-tolerant (HT) and heat-sensitive (HS) genotype: Tuberization is regulated by positive (*StSP6A and StBEL5*) and negative regulators (*StSUT4, StSP5G*, and *StRAP1*). Under high temperature, the positive regulators were relatively stable in the HT genotype as compared to the HS genotype. The stability might be attributed by reduced expression of upstream negative regulators, increased DNA demethylation (*StDeMet2, StDeMet3, StDeMet4, StDeMet5, StDeMet6, StDeMet7*, and *StDeMet8*) and active RdDM (DRM and CMT subfamilies) pathway in leaves and stolon samples of HT genotype. On the contrary, the HS genotype has heat-induced upregulation of negative regulators, thereby affecting the process of tuberization. Stronger upregulation of HSFs (*StHSFA5* and *StHSFA8*) was observed in leaves and stolon tissues of HT genotype as compared to HS genotype maintaining the health status of the tolerant cultivar.

DNA methylation and demethylation events are correlated with the gene expression patterns, thus regulating plant development and stress responses (Choi and Sano, 2007). Also, the genetic control of potato tuberization is well-studied. We observed that the expression of positive regulators of tuberization (*StSP6A* and *StBEL5*) was found to be stable in the leaves and stolon tissues in the HT genotype as compared to the HS genotype under high-temperature stress (Figure 8). The stability of the *StSP6A* transcript under high temperature in HT genotype might be due to the reduced expression of upstream negative regulators (*StSUT4, StSP5G*, and *StRAP1*). Heat induced the expression of *StSUT4* in the leaves and stolons of the HS genotype, which might have led to the activation of the *StCOL1*-*StSP5G* regulatory module. Such studies are consistent with the recent finding that elevated temperature caused higher *StCOL1* accumulation and suppressed potato tuberization (Park et al., 2022). Our expression analysis revealed the higher expression of *StSP5G* transcript under high temperature in the HS genotype. However, such enhanced expression of *StSP5G* did not alter the basal transcription of *StSP6A* in stolons of HS genotype. The expression levels of *StSP5G* and *StSP6A* are usually difficult to interpret in vegetative tissues like stolons and tubers (Hancock et al., 2014). In addition, different regulatory pathways operate for photoperiod and temperature-dependent tuberization, suggesting that a complex regulatory network is involved in the regulation of potato tuberization (Park et al., 2022).

Additionally, we observed a spike in *StRAP1* expression (<50-fold) in leaves of HS genotype, which acts to suppress a phloem mobile transcript (*StBEL5*), thereby delaying tuber induction (Banerjee et al., 2006). Other than genetic factors, the role of DNA methylation machinery components was sought in potato tuberization under high temperature. The increased expression of positive regulators of tuberization in HT genotype might be due to increased DNA demethylation and the active RdDM pathway in the stolons. Furthermore, we observed stronger upregulation of *StHSFA5 and StHSFA8* (<3 and <5-fold, respectively) in leaves and (<50- and <30-fold) in stolons of the tolerant genotype as compared to the HS genotype. This may contribute to the better heat stress resilience of the HT genotype (Tang et al., 2016; Ramakrishnan et al., 2022). Altogether, a complex interplay of genetic and epigenetic factors controls potato tuberization under high-temperature conditions.

## Conclusion

Over the last few decades, the mechanisms underlying plant responses to abiotic stresses have been studied in most of the model plants such as Arabidopsis, rice, and tomato. However, only a few studies are available on other important crops such as potato. More detailed studies are needed to shed light on the signaling networks and downstream target genes controlling potato response to heat stress. Importantly, understanding the interplay of genetic and epigenetic factors in plant stress response will provide new avenues for crop improvement. In this work, we report the characterization of DNA methyltransferase and demethylase gene families in the potato. The expression of *C5-MTases* and *DeMets* were studied in the different tissues of heat-tolerant and heat-sensitive genotypes followed by the expression analysis of tuberization genes. We found enhanced expression of *DeMets* and RdDM genes in HT genotype as compared to HS genotype. These results suggest that increased activity of *DeMets* might have contributed to transcriptional activation of tuberization genes wherein *RdDM* stabilized genomic integrity of HT genotype. Overall, this work will open new research directions on the role of DNA methylation machinery in potato tuberization under high temperature, which is important in the context of global temperature rise.

## Data availability statement

The raw data supporting the conclusions of this article will be made available by the authors, without undue reservation.

## Author contributions

MD performed *in silico* analysis, experiments, analyzed data, and wrote the original draft with figures. VR performed *in silico* analysis and experiments. VG performed *in silico* analysis and writing. AK, PS, and VV performed experiments. VKG and SS provided material and discussed data. GZ conceived the idea, edited, and finalized the manuscript. All authors contributed to the article and approved the submitted version.

## Conflict of interest

The authors declare that the research was conducted in the absence of any commercial or financial relationships that could be construed as a potential conflict of interest.

## Publisher's note

All claims expressed in this article are solely those of the authors and do not necessarily represent those of their affiliated organizations, or those of the publisher, the editors and the reviewers. Any product that may be evaluated in this article, or claim that may be made by its manufacturer, is not guaranteed or endorsed by the publisher.
