# Supplementary material for: The interplay of DNA methyltransferases and demethylases with tuberization genes in potato (Solanum tuberosum L.) genotypes under high temperature
Source: Front Plant Sci. 2022 Aug 16;13:933740. doi: 10.3389/fpls.2022.933740 (PMC9425917; doi:10.3389/fpls.2022.933740)
Supplement: Supplementary file 1 [file Data_Sheet_1.docx]

**Table S1:** Sequence of the primers used for qPCR in this study

| **S No.** | **Gene name** | **Primer Sequence** |
| --- | --- | --- |
| **1.** | *Stβ-Tubulin-F* | ATGTTCAGGCGCAAGGCTT |
|  | *Stβ-Tubulin-R* | TCTGCAACCGGGTCATTCAT |
| **2.** | *StMET2-11A-F* | GCCATGCAGGCTACTACAACAA |
|  | *StMET2-11A-R* | GGAACATTCAAGTTCTCCTCCG |
| **3.** | *StMET1-4A-F* | CGCAATACGTCTTCCTCATCCA |
|  | *StMET1-4A-R* | TGGTATGTATCAACCCCACTGT |
| **4.** | *StDNMT2-8A-F* | CCACTGCAATTTGGACAGCCAT |
|  | *StDNMT2-8A-R* | CAGCAGCTGTTCCCTTTCCATT |
| **5.** | *StCMT3-8A-F* | ATCGGAACAGCATTTCACCTGA |
|  | *StCMT3-8A-R* | CTTATCCTCCGCCGTCGTTGTC |
| **6.** | *StCMT3-1A-F* | AGTTCTTGAAGCCAAGGTTCGT |
|  | *StCMT3-1A-R* | GTCCATATGCCCCAGCAACCAT |
| **7.** | *StCMT3-12A-F* | AGGATGTGGAGCAATGTCCACA |
|  | *StCMT3-12A-R* | ACTGGCATACTCATTCCTCACC |
| **8.** | *StDRM3-10A-F* | ATGTGAAGCCAAACCTGAAGGA |
|  | *StDRM3-10A-R* | CCAAAGCCAATCATCGGTTTGG |
| **9.** | *StDRM1-2A-F* | GGCCATCGAAGAGTCTCCTGAA |
|  | *StDRM1-2A-R* | AGTCTGAGTCAGAGGACCAACT |
| **10.** | *StDRM2-4A-F* | AGATGATAGTTGGTCCTCGGAC |
|  | *StDRM2-4A-R* | CACATCTCTCCATCGCTATGGA |
| **11.** | *StDNMT2-8B-F* | AATTGGTTGCCGATGATTGGTG |
|  | *StDNMT2-8B-R* | AGAGACAGACCCTGCAGTGAAG |
| **12.** | *StDemethylase1-F* | GAGAGACACAGCATCCACAGAC |
|  | *StDemethylase1-R* | ATACCAGATGAAGTGCTGCAGT |
| **13.** | *StDemethylase2-F* | GCAGCACAGTTCAATGGGAACA |
|  | *StDemethylase2-R* | TAGGTCCTCCTTGCTCTTTGGA |
| **14.** | *StDemethylase3-F* | TGTTGTCGGCAGAATCTGCACA |
|  | *StDemethylase3-R* | TCTGGAACTTGCTGGACATTGC |
| **15.** | *StDemethylase4-F* | TGTCGAGGACAGGGTAATGTTC |
|  | *StDemethylase4-R* | CCAAGCCCAGGAATACTCAACA |
| **16.** | *StDemethylase5-F* | GTTCCATGGACGGGTGATATCA |
|  | *StDemethylase5-R* | GCAGAGCTCGATAAGTTGTCTG |
| **17.** | *StDemethylase6-F* | CTTCCAAATCCGTGCGTTTGAC |
|  | *StDemethylase6-R* | TGCATCTTCTGCGGATCTCATC |
| **18.** | *StDemethylase7-F* | ATGCACCTGTAGACTCCATTGA |
|  | *StDemethylase7-R* | CCATTTTCGAGGAGCCGTTGAT |
| **19.** | *StDemethylase8-F* | CAACCTGGGAATGTGTTCTAGC |
|  | *StDemethylase8-R* | AGCTCTCTCAAGTACTCCAAGC |
| **20.** | *StSP6A-F* | TTGGTCGTGTGATAGGTGATGT |
|  | *StSP6A-R* | ATCGTCCCCTCCAATATGAACC |
| **21.** | *StBEL5-F* | CGGCAGTAAGTTTGCACGATC |
|  | *StBEL5-R* | GAAACGCCAATTGAGACGCCA |
| **22.** | *StRAP1-F* | ATGTGGACGATGGGAAGCTC |
|  | *StRAP1-R* | TGCACCTCCATTATCAGCCTC |
| **23.** | *StHSFA5-F* | CAACTGCCAACAAGCAAGTG |
|  | *StHSFA5-R* | TTCTCTCGTCTTGCTCGTCGTA |
| **24.** | *StHSFA8-F* | GTTCAGTTTTTCCAGCCCAAGG |
|  | *StHSFA8-R* | CTCTGCAGCTTCTTCATGAGTG |
| **25.** | *StSP5G-F* | ATGTGCCTTGAGGCCTTCA |
|  | *StSP5G-R* | GGATATCTGTGACCAGCCAGTG |
| **26.** | *StSUT4-F* | AAGTGCACAAGTCGGTTCGG |
|  | *StSUT4-R* | GACAAACGCCGCTATAGCACG |

**Table S2**: Information of C5-MTase genes in the 5 tested species

| **Latin name of species** | **Gene name** | **Gene ID** |
| --- | --- | --- |
| *Arabidopsis thaliana* | *AtMET1* | AT5G49160 |
|  | *AtMET2a* | AT4G14140 |
|  | *AtMET2b* | AT4G08990 |
|  | *AtMET3* | AT4G13610 |
|  | *AtCMT1* | AT1G80740 |
|  | *AtCMT2* | AT4G19020 |
|  | *AtCMT3* | AT1G69770 |
|  | *AtDRM1* | AT5G15380 |
|  | *AtDRM2* | AT5G14620 |
|  | *AtDRM3* | AT3G17310 |
|  | *AtDNMT2* | AT5G25480 |
| *Glycine max* | *GmMET1* | Glyma04g36150 |
|  | *GmMET2* | Glyma06g18790 |
|  | *GmCMT1* | Glyma01g01120 |
|  | *GmCMT3* | Glyma11g08861 |
|  | *GmCMT4* | Glyma16g17720 |
|  | *GmDRM1* | Glyma02g04060 |
|  | *GmDRM2* | Glyma05g08740 |
|  | *GmDRM3* | Glyma07g36081 |
|  | *GmDRM4* | Glyma17g04254 |
|  | *GmDRM5* | Glyma19g00250 |
| *Oryza sativa* | *OsMET1* | LOC_Os03g58400 |
|  | *OsMET2* | LOC_Os07g08500 |
|  | *OsCMT1* | LOC_Os03g12570 |
|  | *OsCMT2* | LOC_Os05g13780 |
|  | *OsCMT3* | LOC_Os10g01570 |
|  | *OsDRM1* | LOC_Os11g01810 |
|  | *OsDRM2* | LOC_Os03g02010 |
| *Solanum lycopersicum* | *SlMET1* | Solyc11g030600 |
|  | *SlCMT2* | Solyc12g100330 |
|  | *SlCMT3* | Solyc01g006100 |
|  | *SlCMT4* | Solyc08g005400 |
|  | *SlDRM1* | Solyc02g062740 |
|  | *SlDRM2* | Solyc04g005250 |
|  | *SlDRM3* | Solyc10g078190 |
|  | *SlDNMT2* | Solyc08g067070 |
| *Zea mays* | *ZmMET1* | GRMZM2G334041 |
|  | *ZmMET2* | GRMZM2G333916 |
|  | *ZmCMT1* | GRMZM2G025592 |
|  | *ZmCMT2* | GRMZM2G005310 |
|  | *ZmDRM1* | GRMZM2G092497 |
|  | *ZmDRM2* | GRMZM2G137366 |
|  | *ZmDRM3* | GRMZM2G065599 |
|  | *ZmDNMT2* | GRMZM2G157589 |

**Table S3:** Information of DNA demethylase genes in the four tested species

| **Latin name of species** | **Gene name** | **Gene ID** |
| --- | --- | --- |
| *Arabidopsis thaliana* | *AtDME* | AT5G04560 |
|  | *AtDML2* | AT3G10010 |
|  | *AtDML3* | AT4G34060 |
|  | *AtROS1* | AT2G36490 |
| *Cucumis sativus* | *CsDME* | Cucsa.308950 |
|  | *CsDML3* | Cucsa.385370 |
|  | *CsROS1a* | Cucsa.083110 |
|  | *CsROS1b* | Cucsa.378580 |
| *Glycine max* | *GmDME* | Glyma.20G188300 |
|  | *GmROS1a* | Glyma.03G190800 |
|  | *GmROS1b* | Glyma.10G065900 |
|  | *GmROS1c* | Glyma.13G151000 |
| *Solanum lycopersicum* | *SlDME* | Solyc11g007580 |
|  | *SlDML3* | Solyc03g123440 |
|  | *SlROS1a* | Solyc10g083630 |
|  | *SlROS1b* | Solyc09g009080 |
